# Linking COVID-19 and Acute Pancreatitis Through the Pathogenic Effects of the SARS-CoV-2 S Protein Subunit 1 on Pancreatic Stellate Cells and Macrophages

**DOI:** 10.1093/function/zqac009

**Published:** 2022-02-26

**Authors:** J P Neoptolemos, W Greenhalf

**Affiliations:** Department of General, Visceral and Transplantation Surgery, University of Heidelberg, Heidelberg, Baden-Württemberg 69120, Germany; Liverpool GCPLab Facility, University of Liverpool, Liverpool, L7 8TX, UK

**Keywords:** acute pancreatitis, SARS-CoV-2, COVID-19, calcium signalling, pancreatic stellate cells, macrophages

## A Perspective on “SARS-CoV-2 S elicits Ca2 + influx-dependent Ca2 + signals in pancreatic stellate cells and macrophages in situ”

Since the early 1970’s Ole Peterson has been at the forefront of many of the major scientific discoveries that have moulded our understanding of pancreatic acinar cell physiology and of the pathophysiology leading to acinar cell necrosis and acute pancreatitis.^1^ Pulse chase studies in the 1970s identified exocytosis as the key secretory pathway in pancreatic acini, occurring in response to physiologic concentrations of cholecystokinin or acetylcholine. This process involves the transport of proteins from the endoplasmic reticulum to the Golgi complex, concentration within condensing vacuoles and zymogen granules, and release into the extra-acinar lumen at the apical plasma membrane. Subsequent studies showed that supramaximal secretagogue stimulation resulted in blockade of apical exocytosis and release of intracellular granules from the baso-lateral plasma membrane into the intercellular space. This was shown to be a key pathogenic mechanism in the early evolutionary stages of acute pancreatitis. Normal exocytosis is governed by local cytosolic Ca^2+^ spikes in the granular apical region. The Ca^2+^ is released from intracellular stores in the endoplasmic reticulum via inositol trisphosphate and ryanodine receptors, and acid stores via the two-pore channel and ryanodine receptors. Apical cytosol Ca^2+^ spiking also causes increased mitochondrial ATP production, to provide the energy source driving the mechanism of exocytosis.^1^

Pathogenic signals including fatty acids, alcohol, bile acids, and physical pressure result in sustained cytosolic Ca^2+^ overloading. The consequences are direct trypsinogen activation within the zymogen granules with intra-cytosolic release of trypsin, as well as release into the intercellular space. There is also mitochondrial Ca^2+^ overload with reduced ATP formation. These processes combine to produce acinar cell necrosis and an ever-expanding surrounding focus of tissue destruction. This is amplified by activation of both pancreatic stellate cells (by released kallikrein converted to bradykinin acting on the B2 receptor) generating toxic nitric oxide levels, and activation of resident macrophages (by released ADP and ATP acting on purinergic receptors) generating a series of cytokines including tumour necrosis factor-α. These processes result in further recruitment of circulating inflammatory cells into the foci of developing pancreatic necrosis. In clinically severe acute pancreatitis, the excessive inflammatory cell activation triggers an uncontrolled systemic anti-inflammatory response leading to multi-organ failure, especially affecting the lungs and kidneys.

In this latest work produced by Ole Petersen along with his longstanding co-workers Julia and Oleg Gerasimenko we are shown new insights into the pathogenic effects of the SARS-CoV-2 on pancreatic lobular cells.^2^ The link between SARS-CoV-2 and pancreatitis was identified very early on in the COVID-19 pandemic but it's true significance remains unclear.^3^ The nature of the link remains contentious, with some groups suggesting that the viral infection was not directly linked to the pancreatitis and even that many cases of pancreatitis were spurious since the raised amylase seen in patients with COVID-19 was mostly the result of a non-specific manifestation of shock in a critical illness. It has been open to debate whether this is direct viral damage of pancreatic cells or whether the damage is an indirect consequence resulting from the immune response.^4^ This is of considerably more than academic interest, as it has been proposed that although diagnosis of acute pancreatitis is relatively rare in COVID-19, pancreatic inflammation may be a critical factor in COVID-19 associated deaths. In the context of a critically severe illness represented by COVID-19 the revised 2012 Atlanta Criteria for defining the diagnosis and level of severity may be misinterpreted and/or overinterpreted. This is illustrated by the COVID PAN collaborative study in which the results of serum amylase and/or lipase levels were not reported, and there was also no mention of the characteristic diagnostic findings of acute pancreatitis on contrast-enhanced computed tomography and magnetic resonance imaging.^5^ Which leaves us with a pressing need to identify and characterise the specific mechanisms associated with the initiation of pancreatitis by SARS-CoV2 and how the severity is determined.

SARS-CoV-2 tropism towards distinct tissues is governed by cellular factors expressed on target cells. Viral entry requires attachment of the spike protein to angiotensin-converting enzyme 2 (ACE2).^6^ The transmembrane serine protease 2 (TMPRSS2) is also required for cleavage of sites S1 and S2 for membrane fusion and receptor mediated endocytosis.^6^ ACE2 and TMPRSS2 have both been identified in pancreatic endothelial pericytes and also in pancreatic ductal cells.^7,8^ Expression of ACE2 and TMPRSS2 in human acini have also been found but this seems to be at low levels and patchy in distribution.^7,8^ Genetic variants of TMPRSS2 may account for differential susceptibility to SARS-CoV-2 infection and COVID-19 disease severity.^9^

SARS- CoV-2 is also able to cause diffuse severe endothelial inflammation, and microvascular thrombosis in several anatomical sites, leading to diffuse microischaemia, an established cause of acute pancreatitis.^10^ Nevertheless ischaemia can cause oedema of the pancreas along with raised circulating enzyme levels without necessarily causing pancreatitis, which requires acinar cell necrosis as a defining characteristic. Post-mortem examinations have detected SARS-CoV-2 nucleocapsid protein in pancreatic exocrine cells, raising the question whether infection of pancreatic ducts and acinar cells is associated not just with elevated amylase and lipase levels elevations in patients with SARS-CoV-2-associated pneumonia, but also acute oedematous pancreatitis.^8^ None of the post-mortem studies on the four patients who died from COVID-19 however with pancreatic exocrine 2 nucleocapsid protein expression had any gross pathological or histological features of pancreatitis.^8^

Nevertheless, we can hypothesize three routes by which SARS-CoV-2 might cause acute pancreatitis: (i) direct infection of pancreatic acinar cells, (ii) infection of pancreatic ductal cells with subsequent lobular disruption, and (iii) microischaemia leading to pancreatic acinar necrosis. The paper by Gerasimenko and colleagues is a breakthrough in demonstrating that there is a direct action of SARS-CoV2 on the pancreatic parenchymal stellate cells leading to the production of IL-18 and activation of resident pancreatic macrophages.^2^ Whether this is sufficient to initiate pancreatic acinar cell necrosis of itself is unclear at the present time, but may be contributory along with the aforementioned hypothetical mechanisms. Even so pancreatic IL-18 activated macrophages could promote clinical deterioration in otherwise critically ill patients. It is also significant that in the Gerasimenko model spike protein, or even just part of the spike protein, could cause pancreatitis in a susceptible individual, perhaps giving a mechanism to explain reports of acute pancreatitis following vaccination.
